# Human immunodeficiency virus associated pulmonary conditions leading to hospital admission and the pulmonary rehabilitation services received by patients at two central hospitals in Harare

**DOI:** 10.1186/s13104-018-3525-0

**Published:** 2018-06-25

**Authors:** C. Tadyanemhandu, C. Mupanda, J. Dambi, M. Chiwaridzo, V. Chikwasha, S. Chengetanai

**Affiliations:** 1 Department of Rehabilitation, College of Health Sciences, University of Zimbabwe, Avondale, PO Box AV 178, Harare, Zimbabwe; 2 Department of Community Medicine, College of Health Sciences, University of Zimbabwe, Avondale, PO Box AV 178, Harare, Zimbabwe; 3 Division of Basic Medical Sciences, Faculty of Medicine, National University of Science and Technology, Ascot, PO Box AC 939, Bulawayo, Zimbabwe

**Keywords:** Pulmonary manifestations, Physiotherapy practice, Pulmonary rehabilitation, HIV/AIDS, Tuberculosis, Chronic lung diseases

## Abstract

**Objective:**

Use of highly active antiretroviral therapy has led to marked reductions in the incidence of HIV-associated opportunistic infections but has had comparatively less impact on the incidence of some pulmonary diseases. This study was done to determine the pulmonary conditions leading to hospital admissions in people living with HIV/AIDS at two central hospitals in Zimbabwe and the pulmonary rehabilitation intervention received.

**Results:**

A total of 92 participants were recruited of which 60 (65.2%) were females. The mean age of the participants was 41.3 years (SD = 9.1). The most common pulmonary condition leading to hospital admission was tuberculosis in 53 (57.6%). About 52 (56.6%) of the participants suffered from pulmonary complications in the last 6 months, 48 (92.3%) were admitted and 26 (50.0%) of the participants received physiotherapy treatment during their admission. None of the participants indicated that they once attended an outpatient pulmonary rehabilitation clinic. Respiratory complication is one of the leading causes of morbidity associated with HIV but no pulmonary rehabilitation services are being offered to these patients. There is need for introduction of pulmonary rehabilitation programs for people living with HIV/AIDS in the current setting.

## Introduction

Zimbabwe has the sixth highest human immunodeficiency virus (HIV) prevalence in sub-Saharan Africa at 13.5%, with 1.3 million people living with HIV and AIDS (PLWHA) in 2016 with about 74% of the adults on antiretroviral treatment [1]. Use of highly active antiretroviral therapy (HAART) has led to marked reductions in the incidence of HIV-associated opportunistic infections (including Pneumocystis pneumonia, cytomegalovirus disease and Mycobacterium avian complex) and tumours (including Kaposi sarcoma) [2]. However, the impact has been relatively less on the incidence of some pulmonary disease which includes bacterial pneumonia and tuberculosis (TB) [2–4].

Pulmonary diseases are a significant cause of morbidity and mortality in HIV infected individuals and the infections and complications associated with HIV are broad [5]. Besides the infectious pulmonary complications which include TB and pneumonia, chronic lung disease (e.g. chronic obstructive pulmonary diseases [COPD], bronchiectasis) is an increasingly recognised but poorly understood complication in PLWHA which requires further research to determine optimal screening, diagnostic and treatment strategies in different populations [6–8].

One of the long term treatment strategies being implemented in PLWHA with chronic pulmonary conditions is pulmonary rehabilitation [9, 10]. Pulmonary rehabilitation (PR) is an evidence-based, multidisciplinary, comprehensive intervention which is advocated for patients with chronic respiratory diseases who are symptomatic and often have progressively restricted daily-life activities [11, 12]. PR is recommended in patients with a significant functional limitation due to the pathological state caused by many patho-anatomical changes during the healing process of HIV associated pulmonary complications, affecting the quality of life of patients [12]. In a recent cross-sectional study done in Zimbabwe on the HIV associated chronic lung diseases in children and adolescents, high resolution computed tomographic findings showed that obliterative bronchiolitis was the major cause of chronic pulmonary disease in this cohort [6]. Against this background, the study was done to determine the pulmonary complications leading to hospital admissions in adults living with HIV/AIDS at two central hospitals in Harare and the pulmonary rehabilitation intervention received to reduce the impact of the symptoms. The information obtained from this study will inform a bigger study which will be focusing on implementation of pulmonary rehabilitation programs in people living with HIV/AIDS and determining the efficacy and effect of the treatment on quality of life in Zimbabwe.

## Main text

### Materials and methods

#### Study design, setting and participants

A descriptive cross sectional study using a survey questionnaire was done in January and February 2016 at two central hospitals in Harare. PLWHA admitted in the adult medical wards at the two central hospitals with a diagnosis by the attending physician of a pulmonary complication were recruited to participate in the study. Consecutive sampling was used to recruit the patients who met the inclusion criteria.

#### Study instrument and procedure

A self-administered questionnaire that consisted of three sections was used to collect data. The questionnaire was developed through literature search [13]. Data collected in the first section of the survey questionnaire included demographic information such as age, gender, marital status, occupation, educational level, employment, duration on ART, duration of infection, the most recent CD4 count.

The second section collected data on the HIV associated pulmonary complication which had led to the admission both infectious and non-infectious diseases, the respiratory symptoms the participants were presenting with and the comorbidities present. The third section of the survey questionnaire gathered information on any respiratory condition the participant had suffered from in the previous 6 months before the current admission, the chest physiotherapy intervention the participant received and the outpatient pulmonary rehabilitation services offered to the participants.

The questionnaire was researcher administered. One of the authors (CM) went through the admission ward books of the medical wards in the two hospitals to identify the participants who met the inclusion criteria. After identification of the patient name and hospital bed, the researcher approached the participant and the objectives and purpose of the study was explained to the participant before signing of informed consent. After signing of the informed consent, the researcher accessed the patient’s notes to view the clinical history of the participant. The researcher interviewed the participants to elicit information regarding the following: their current respiratory problems, previous respiratory episodes in the past 6 months and the chest physiotherapy they received previously including the outpatient pulmonary rehabilitation services received. All the information was documented by the researcher on the questionnaire.

#### Data analysis

Data was analysed using SPSS (Version 25). Descriptive statistics were used; mean ± SD for parametric variables and median (IQR) for non-parametric variables whilst categorical data was reported as frequencies. Independent-T test assuming unequal variances was used to compare differences between age and gender whilst the Mann–Whitney U test checked for significant differences in the sum ranks of the CD4 count and duration of infection by gender.

### Results

#### Demographics of the participants

A total of 92 participants admitted during the study period were enrolled, of which 60 (65.2%) of the participants were females. The mean age for the participants was 41.3 (SD = 9.1) years. Majority of patients were married constituting 80 (87.0%) of the study participants. In terms of occupation, 50 (54.4%) of the participants were informally employed and 57 (62.0%) had the highest educational level of secondary (Table 1). The CD4 counts were only done in 83 (90.0%) participants. The CD4 count median was 153 cells/mm^3^ (IQR = 105–312). There was no significant difference in the rank sum of the CD4 count by gender (U = 745, p = 0.8). The median infection duration was 7 years (IQR = 5–12) with a range of 1–17 years. There was no significant difference in the rank sum of the duration of infection by gender (U = 792, p = 0.5). All of the participants were on ART.

**Table 1:** Demographics and clinical information for the patients (n = 92)

Variable	Attribute	n (%)
Sex	Female	60 (65.2)
	Male	32 (34.8)
Marital status	Single	9 (9.7)
	Married	80 (87.0)
	Divorced	3 (3.3)
Educational level	Primary	27 (29.3)
	Secondary	57 (62.0)
	Tertiary	8 (8.7)
Occupation	Informal employment	50 (54.4)
	Formal employment	9 (9.7)
	Unemployed	33 (35.9)
ART	Yes	92 (100.0)

#### Pulmonary complications in people living with HIV/AIDS

The most common pulmonary condition was tuberculosis seen in 53 (57.6%) of the participants and it was more prevalent for CD4 cell count between 100 and 199 cells/mm^3^, followed by bacterial pneumonia in 25 (27.2%) participants, it was most prevalent for CD4 count between 300 and 399 cells/mm^3^ (Table 2). The most common respiratory symptom in the participants was a productive cough in 55 (59.8%) of the participants, 37 (40.2%) of the participants had chest pain and 36 (39.1%) of them complained of breathlessness. About 59 (64.1%) of the participants had comorbidities and diabetes was the commonest, 17 (18.5%).

**Table 2:** Distribution of cases according to pulmonary diagnosis and relation to CD4 count (n = 92)

Pulmonary diagnosis	No of cases with CD4 cells/mm^3^						Total, n (%)
	0–99	100–199	200–299	300–399	> 400	Absent	
TB	17	27	3	3	0	0	53 (57.6)
Bacterial pneumonia	2	2	5	11	1	4	25 (27.2)
PCP	6	4	0	0	0	2	12 (13.0)
Emphysema	1	1	1	1	0	1	5 (5.4)
Kaposi sarcoma	3	0	0	0	0	0	3 (3.3)
Bronchiectasis	1	0	0	1	1	1	4 (4.3)
Chronic bronchitis	1	1	0	1	0	0	3 (3.3)

*TB* tuberculosis, *PCP* Pneumocystis pneumonia

#### Pulmonary rehabilitation of the pulmonary complications of HIV/AIDS

Out of the 92 participants, 52 (56.6%) had a pulmonary complication in the last 6 months from the date of data collection. Of these, 48 (92.3%) of the participants were admitted following a pulmonary complication and 26 (50.0%) of the participants reported to have received chest physiotherapy treatment during their admission. The participants indicated that the chest physiotherapy treatment received during their previous admission consisted of secretion expectoration techniques in 16 (61.5%) of the participants and breathing exercises in 18 (69.2%) of the participants. None of the participants indicated that they once attended an outpatient pulmonary rehabilitation clinic.

### Discussion

The results of our study showed that TB, bacterial pneumonia and Pneumocystis pneumonia were the most common pulmonary complications leading to hospital admission in PLWHA at the two central hospitals. These complications were present in people of wide range of CD4 count but they were most prevalent in people who had a low CD4 count. In a similar study in North India, it was also reported that pulmonary TB and pyogenic pneumonia were present in over a wide range of CD4 count, but their incidence increased as the CD4 count declined [13]. Zimbabwe is the 17th highest TB burden country in the world, and TB is the second leading cause of severe illness and mortality in Zimbabwe [1] and this correlates well with our findings as majority of the participants were admitted into the hospital as a result of TB infection. Unlike in most low and medium income countries where the prevalence of TB is high, in high income countries, community-acquired bacterial pneumonia is more frequent than Pneumocystis pneumonia (PCP) or TB [5]. Participants from our study also presented with the following conditions; bronchiectasis, emphysema and chronic bronchitis, which are classified as chronic respiratory diseases reported to be strongly associated with high prevalence of HIV and TB. There is a transition being noticed in many countries, that of a decrease in burden of infectious diseases but a greatly increased burden of non-communicable diseases (NCDs), resulting in many low and middle income countries facing that double burden of diseases [14, 15]. HIV and TB have been reported to be strongly associated with the presence of chronic respiratory diseases in adults which include COPD and bronchiectasis [5, 8, 16–21]. It has been indicated that there is now a great need for health care providers, researchers, policy makers, stakeholders to put more attention to COPD in order to improve detection, overall proper management and efficient control of COPD in PLWHA [19].

The respiratory sequelae which include productive cough, chest pain and breathlessness which were reported by participants from our study, have been reported in other studies too and these symptoms have been reported to persist in individuals who would have been properly treated for either TB or pneumonia [7, 18, 22]. This shows that the conditions have a long term impact on the quality of life of the people, even after successful treatment of these diseases. Women constituted more than half of the people who were admitted due to a respiratory complication in PLWHA in our study. Our findings concur with what has been reported by UNAIDS [1] that an estimate of 720,000 women in Zimbabwe are living with HIV. The reasons for this high prevalence of HIV in women has been attributed to gender inequality within relationships and marriages as women fail to negotiate use of condoms with their partners and cultural beliefs which prevents women from denying sex when the husband is unfaithful, putting then at a higher biological risk of HIV [23].

Our study also showed the gaps in pulmonary rehabilitation services for PLWHA. Participants reported that they had suffered a respiratory complication which had resulted in admission in the past 6 months but not all participants received chest physiotherapy treatment and none of them ever received outpatient pulmonary rehabilitation services after discharge. The reason for this might be because this service is not regarded as a standard operating procedure for all PLWHA who present with a pulmonary complication. This finding is similar to what has been reported in Uganda that chronic pulmonary diseases caused by TB or COPD carry a large but silent burden of human suffering yet its treatment and rehabilitation for patients are not regarded as a health priority [10]. Wilches et al. [9] indicated that in chronic pulmonary patients like TB patients, although receiving all the pharmacological efforts available, they generally continue having physical limitations as the great inflammatory component causes serious injuries that trigger fibroblastic reaction, fibrosis and chest wall retraction, affecting mobility, which compromises pulmonary expansion. PR programs have been reported to result in improved long term outcomes in patients with chronic lung diseases [9, 24, 25]. Since there is a high prevalence of the chronic respiratory symptoms and pulmonary dysfunctions in PLWHA, this indicates the need for further intervention to reduce the long term impact of these symptoms in patients who would have been successfully treated so as to prevent lung damage caused by the disease. Therefore, there is need to consider pulmonary rehabilitation as the standard of care in PLWHA.

## Limitations of the study and recommendations

Our study was without limitations. The first limitation was on the study design which was a cross sectional study design therefore this resulted in failure to assess cause-effect relationship in the population. The other limitation of the study was reliance on self-reported data from patients and hence inaccuracy issues sometimes affect the quality of the data. The use of only two central hospitals also affect the generalisability of the results. There is need to conduct prospective studies to determine the pulmonary rehabilitation services offered to PLWHA with pulmonary manifestations and determine the impact of the treatment on their quality of life.

## Abbreviations

AIDS: Acquired Immune Deficiency Syndrome
ART: antiretroviral therapy
COPD: chronic obstructive pulmonary diseases
HAART: highly active antiretroviral therapy
HIV: human immunodeficiency virus
NCDs: non communicable diseases
PCP: Pneumocystis pneumonia
PLWHA: people living with HIV/AIDS
PR: pulmonary rehabilitation
TB: tuberculosis
